# Theoretical prediction and validation of cell recovery rates in preparing platelet-rich plasma through a centrifugation

**DOI:** 10.1371/journal.pone.0187509

**Published:** 2017-11-02

**Authors:** Linfeng Piao, Hyungmin Park, Chris Hyunchul Jo

**Affiliations:** 1 Department of Mechanical & Aerospace Enginnering, Seoul National University, Seoul, Korea; 2 Institute of Advanced Machines and Design, Seoul National University, Seoul, Korea; 3 Department of Orthopedic Surgery, Seoul Metropolitan Government–Seoul National University Boramae Medical Center, Seoul National University College of Medicine, Seoul, Korea; University of Pittsburgh, UNITED STATES

## Abstract

In the present study, we propose a theoretical framework to predict the recovery rates of platelets and white blood cells in the process of centrifugal separation of whole blood contained in a tube for the preparation of platelet-rich plasma. Compared to previous efforts to optimize or standardize the protocols of centrifugation, we try to further the physical background (i.e., based on the multiphase flow phenomena) of analysis to develop a universal approach that can be applied to widely different conditions. That is, one-dimensional quasi-linear partial differential equation to describe the centrifugal sedimentation of dispersed phase (red and white blood cells) in continuous phase (plasma) is derived based on the kinematic-wave theory. With the information of whole blood volume and tube geometry considered, it is possible to determine the positions of interfaces between supernatant/suspension and suspension/sediment, i.e., the particle concentration gradient in a tube, for a wide range of centrifugation parameters (time and acceleration). While establishing a theory to predict the recovery rates of the platelet and white blood cell from the pre-determined interface positions, we also propose a new correlation model between the recovery rates of plasma and platelets, which is found to be a function of the whole blood volume, centrifugal time and acceleration, and tube geometry. The present predictions for optimal condition show good agreements with available human clinical data, obtained from different conditions, indicating the universal applicability of our method. Furthermore, the dependence of recovery rates on centrifugal conditions reveals that there exist a different critical acceleration and time for the maximum recovery rate of platelets and white blood cells, respectively. The other parameters such as hematocrit, whole blood volume and tube geometry are also found to strongly affect the maximum recovery rates of blood cells, and finally, as a strategy for increasing the efficiency, we suggest to dilute the whole blood, increase the whole blood volume with a tube geometry fixed.

## Introduction

For a few past decades, we have seen the increasing interest and advances in clinical applications of platelet-rich plasma (PRP) to various fields of plastic surgery, dentistry, orthopedics, sports medicine and so on [1–5]. These, in general, require the treatment of chronic wounds and/or muscle injuries, which can be greatly benefited from the positive potentials of PRP in the tissue healing and bone regeneration [4, 6–10]. In order to fully utilize the functionalities of PRP that make these applications promising, it is required to maximize the concentration of platelets (and/or white blood cells, although the beneficial effects from the inclusion of leukocytes are still under a debate), which plays a critical role in releasing growth factors, cytokines and proteinases [8, 9, 11–13]. Thus, many previous studies have tried to optimize (or standardize) the protocols to prepare the platelet-rich plasma so far [14–17], to name some, while various commercial products to produce PRP have been introduced to the market and tested as well [18–20]. Since the quality and functionality of PRP are strongly dependent on the protocol used for its preparation, however, the wide variations in the reported conditions to prepare PRP, such as centrifugal acceleration and time, amount of volume of blood, and the type of anticoagulant platelet agonist, make it very difficult to compare the subsequent results fairly. Thus, a systematic relation between the preparation condition and the concentration of platelets (and/or white blood cells) is sorely required to clarify the clinical benefits (biological effects) of PRP. This issue is considered to be much more significant from the fact that the recovery rate of platelets from the commercial automated system, typically of high cost, is relatively lower (about 40–60%) than expected.

**Fig 1:**
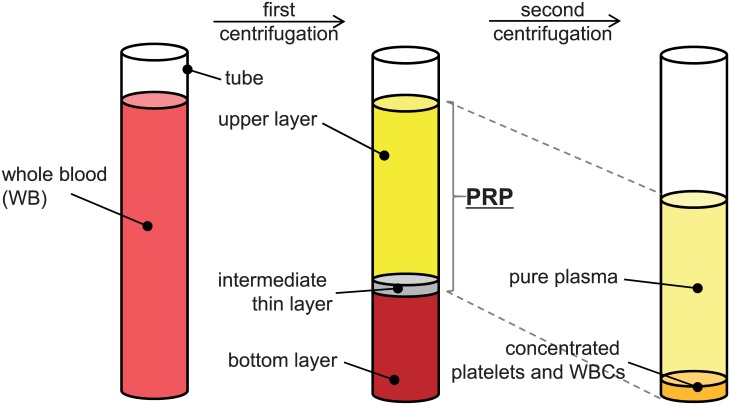
Two-step process of centrifugal separation of the whole blood in a tube for the preparation of platelet-rich plasma (PRP).

In general, a PRP preparation involves sequential steps of blood collection, centrifugation to separate and recover the platelets, and activation of the platelets. The centrifugation step, which is the main interest of the present study, consists of the first stage to separate red blood cells (RBCs) and the second one to concentrate platelets [15–17, 21]. As shown in Fig 1, a whole blood (WB) is initially collected in a tube (with anticoagulants) and the first centrifugation is carried out at a constant speed to separate the RBCs from the whole blood. After this process, the WB is separated into three layers: an upper layer that is mostly occupied with plasma and platelets, an intermediate thin layer including a small amount of platelets and white blood cells (WBCs), and a bottom layer where most of the RBCs is packed. A typical PRP is usually obtained by transferring the upper and intermediate layers to an empty tube. The second centrifugation is then applied to concentrate the platelets and WBCs in the PRP. Thus, it is evident that the final properties of the PRP and efficiency of the whole procedure are strongly subject to the centrifugal conditions such as time (*t*_*c*_) and speed (or centrifugal acceleration, *a*_*c*_) [16, 22], not to mention the influence on the maximized recovery of intact platelets (and/or WBCs).

As we have mentioned above, many previous studies have tried to find out the optimal condition of centrifugation for maximizing the recovery of platelets during PRP preparation [14–16, 21–24]. For example, Slichter & Harker (1976) [22] showed that the single-step centrifugation of WB at a centrifugal acceleration of *a*_*c*_ = 1,000*g* (*g* is the gravitational acceleration) for *t*_*c*_ = 9 minutes provides the maximum recovery rate (up to 89%) of platelets. It was also reported that the shorter centrifugal time could be achieved by applying higher acceleration while the platelet viability is reduced significantly when the acceleration becomes greater than 3,000*g*, being applied for 20 minutes. Kahn *et al*. (1976) [14] explored the most efficient set of centrifugal conditions at relatively higher accelerations (*a*_*c*_ = 1,614*g*–3,731*g*). They showed that the recovery rate of platelets does not appreciably increase when the spin time is longer than 8 minutes and the applied acceleration is larger than 2,324*g*. Considering the loss of platelet integrity together, thus several studies [15, 16, 21, 23] recently reported optimal centrifugation conditions at relatively smaller accelerations (*a*_*c*_ < 1,000*g*) to achieve the maximum recovery of platelets from the first stage of spin. Unfortunately, it is found that the results from these studies are inconsistent with each other and show a wide scattering in the optimal centrifugal conditions and the achievable recovery of platelets. Araki *et al*. (2012) [15], for example, obtained 80%–90% maximum recovery rate of platelets with *a*_*c*_ = 230*g*–270*g* and *t*_*c*_ = 10 minutes, while Amable *et al*. (2013) [23] reported about 87.7% platelet recovery rate with *a*_*c*_ = 300*g* and *t*_*c*_ = 5 minutes. Jo *et al*. (2013) [16] achieved a quite high platelet recovery rate of 92% at *a*_*c*_ = 900*g* and *t*_*c*_ = 5 minutes, and Perez *et al*. (2013) [21] obtained about 80% maximum recovery rate of platelets with *a*_*c*_ = 100*g* and *t*_*c*_ = 10 minutes. On the other hand, it is also necessary to solidify our understanding on the dependence of WBC recovery on centrifugal conditions, in addition to clearing the clinical role of WBCs [9]. Araki *et al*. (2012) [15] obtained about 25% recovery of WBCs and 70%–80% of platelets with *a*_*c*_ = 70*g* and *t*_*c*_ = 10 minutes. Perez *et al*. (2014) [24] showed the maximum recovery of WBCs (∼10%) and platelets (∼80%) with *a*_*c*_ = 100*g* and *t*_*c*_ = 10 minutes. It is noted that they all showed the recovery of WBCs is reduced to almost zero when the centrifugal acceleration is larger than about 800*g*.

Now, it is clear that the widely scattered centrifugal conditions and resulting recovery of platelets and WBCs in PRP make it necessary to develop a universal protocol to optimize the PRP preparation method, which can be benefited significantly from a systematic analysis based on a physical theory underlying the separation process, i.e., multiphase flow phenomena. Considering the plasma and platelets (and/or WBCs) as continuous and dispersed phases, respectively, it is possible to utilize our knowledge in particle sedimentation. Perez *et al*. (2013) [21], sharing the same idea as ours, suggested a mathematical model to predict the recovery of platelets, based on the Stokes’ law that describes a settling velocity of a single spherical particle in an unbounded environment. While the effect of backflow of the cell suspension has been included in their model, we think that more improvements are required to consider the influences from particle-particle interactions and other operating conditions such as confined tube geometry and initial volume of whole blood.

Therefore, in the present study, we propose a new theoretical model to predict the recovery rates of the platelets and WBCs in PRP prepared by spinning whole blood contained in a tube. To solidify the physical background of the developed model, we apply the kinematic wave theory to estimate the volume fractions of each phase, i.e., plasma, platelets and WBCs, as a function of centrifugation time, acceleration, tube geometry, and the initial state of whole blood. To achieve this, we also perform a dimensional analysis to decide principal non-dimensional parameters that determine the recovery rate. The proposed model is applied to a wide range of parameters including volume of WB, hematocrit (volume fraction of RBC in WB), centrifugal time and acceleration, which is at the same time fully validated with available experimental (human clinical) data. Further discussions on the dependency of PRP composition on various parameters are given, and the results found in this study are expected to formulate practical guidelines for optimizing the PRP preparation.

## Establishment of theoretical model

### One-dimensional kinematic wave for the centrifugal sedimentation

**Fig 2:**
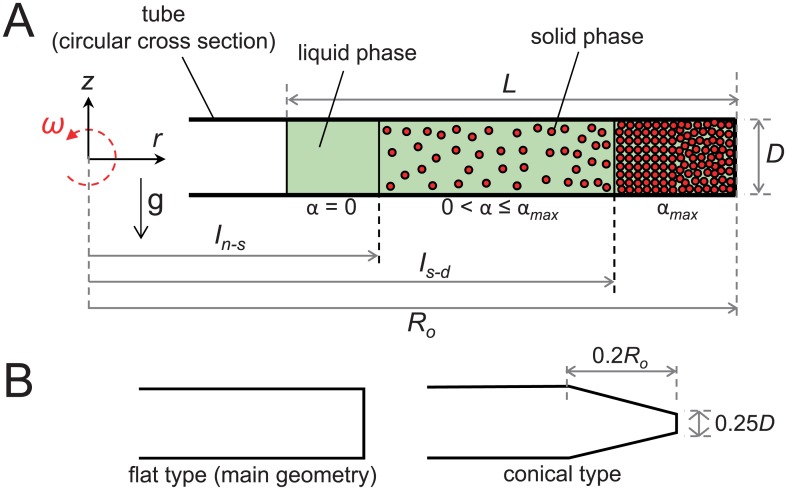
Definition of modeled problem. A: One-dimensional description of centrifugal sedimentation of particles in a rotating tube with a constant cross-sectional area. After spinning for a certain time duration, the concentration zones are classified as a clear liquid (*α* = 0), the retarded settling zone (0 < *α* ≤ *α*_*max*_) and the packed bed (bottom layer) (*α* = *α*_*max*_). Here, *α* is the volume fraction of solid phase, *α*_*max*_ is the maximum concentration of the solid phases (or particles) in the packed bed, *L* indicates height of solid-liquid mixture, *D* is diameter of the tube and *R*° is distance between the center of rotation and tube bottom. *I*_*n* − *s*_ and *I*_*s* − *d*_ denote the position of the interfaces between supernatant/suspension and suspension/sediment, respectively. B: Two types of tube geometry considered in the present study.

In the present study, we start from the idea that the packing of RBCs and WBCs at the bottom of the tube during the centrifugal separation of a whole blood can be considered as a centrifugal sedimentation of particles (dispersed phase) in a liquid (continuous phase). Especially, the one-dimensional kinematic wave theory is a very useful tool for our analysis, which is well known for its capability to determine gradient of particle concentration (i.e., interfaces between supernatant, suspension and sediment) in a tube [25–30]. Here, we consider the RBCs, WBCs and platelets as spherical solid particles of uniform diameter (*d*_*RBC*_, *d*_*WBC*_, and *d*_*PLT*_, respectively) and density (*ρ*_*RBC*_, *ρ*_*WBC*_, and *ρ*_*PLT*_, respectively), and the plasma as a liquid phase (density, *ρ*_*plas*_ and dynamic viscosity, *μ*_*plas*_). In Fig 2A, we have illustrated the present problem of spinning (at a constant angular velocity of *ω*) a tube that contains an incompressible mixture (i.e., initial whole blood, WB) of solid and liquid phases. Considering that a typical centrifugal acceleration (acting along the *r*-direction) applied for the blood separation is as large as ***O***(10^2^–10^3^)*g*, the effect of gravitational field acting in *z*-direction is assumed to be negligible, and thus the particle movement along *r*-direction (i.e., one-dimensional motion) is considered to be dominant [26, 28, 29]. For the shape of tube bottom, we consider a flat geometry, simplifying that of a typical plain tube used in common [16]. As we will explain below, our prediction model can be applied to different tube geometry, i.e., the effect of tube geometry can be evaluated, and thus additional tube bottom shape is considered for the comparison (Fig 2B).

Now, we will constitute the mass and momentum conservation equations for the present problem. For a one-dimensional solid-particle suspension in a long tube, like the present case shown in Fig 2A, the unsteady continuity (mass conservation) equations for solid phase and mixture of liquid-solid phase are written as:

$$\begin{array}{c} \frac{\partial \alpha }{\partial t}+\frac{\partial j_{s}}{\partial r}=0 \end{array}$$ (1)

and

$$\begin{array}{c} \frac{\partial j}{\partial r}=0, \end{array}$$ (2)

respectively, where *α*(*t*, *r*) is the volume fraction of the solid phase (i.e., particle concentration), *j*(*t*, *r*) and *j*_*s*_(*t*, *r*) denote the volume flux density [*ms*^−1^] of liquid-solid mixture and solid phase, respectively. Total volume flux density is then calculated as the sum of solid (*j*_*s*_) and liquid (*j*_*l*_) volume flux densities. Based on drift-flux model [31], the volume flux density of a solid phase in the reference frame moving at the volume flux density (*j*) of liquid-solid mixture, that is the drift flux *j*_*sl*_, is expressed as:

$$\begin{array}{c} j_{sl}=\alpha v_{s}-\alpha j, \end{array}$$ (3)

where *v*_*s*_(*t*, *r*) denotes the velocity of a solid particle. Since the volume flux density *j*_*s*_ is related to the concentration *α* such as *v*_*s*_ = *j*_*s*_/*α*, then the Eq 3 can be rewritten as:

$$\begin{array}{c} j_{sl}=j_{s}-\alpha j. \end{array}$$ (4)

Considering that the settling velocity of a particle should change along the *r*-direction, on the other hand, the momentum conservation equation for the solid particle motion relative to liquid phase can be reduced to a relationship between the drift flux (*j*_*sl*_) and the settling function that is associated with the concentration *α*, as shown below [26, 28]:

$$\begin{array}{c} j_{sl}=r\frac{\omega^{2}}{g}f_{bk}\left(\alpha \right). \end{array}$$ (5)

Here, the function *f*_*bk*_(*α*) is defined as a Kynch batch flux density function [28] that describes the effect of existence of adjacent particles (i.e., effects from multiple particles are considered) on the settling (terminal) velocity of a single particle (*u*_∞_) through a stationary liquid, determined by the Stokes’ law such as $u_{\infty }=\left(\rho_{s}-\rho_{l}\right)d_{s}^{2}g/18\mu_{l}$ (subscripts ‘s’ and ‘l’ denote solid and liquid phase, respectively), where *ρ* and *μ* denote the density and viscosity, respectively, and *d*_*s*_ is the size of the particle. That is, it accounts for the retarded settling due to the interactions between particles and a backflow from the bottom of the vessel. For the function *f*_*bk*_, we use the well-known Richardson-Zaki model [32], which has been widely used to similar problems to the present one [26, 28, 31]. The actual model is given as:

$$\begin{array}{c} f_{bk}=\{\begin{array}{cc} -u_{\infty }\alpha {\left(1-\alpha \right)}^{m} & \;\text{for}\;0<\alpha <\alpha_{max}\\ 0 & \;\text{otherwise} \end{array}, \end{array}$$ (6)

where *m* is an index (or exponent) that is a function of the particle Reynolds number (*Re*_*p*_) [31, 32]. For a spherical solid particle, *m* = 5 is typically used when *Re*_*p*_ is smaller than 0.2 [26]. For the present study, the particle Reynolds number (for RBCs and WBCs) is *Re*_*p*_∼ ***O***(10^−2^–10^−1^). This function also indicates that the settling is terminated at a limiting value of *α* = *α*_*max*_ which is the maximum concentration of particles in the packed bed, i.e., in the bottom layer (see Fig 2A). In the present analysis, we use the maximum concentration value of *α*_*max*_ = 0.8, considering the deforming property of the blood cells [27, 29, 33].

As introduced above, Perez *et al*. (2013) [21] also suggested a theoretical prediction of cell recovery, based on the modified settling velocity of a particle under centrifugation. They have corrected the settling velocity (*u*_∞_) of a single particle (i.e., RBC) to consider the effect of backflow as:

$$\begin{array}{c} u_{s}=Ku_{\infty }\left(1-\alpha_{BL}\right)/\alpha_{BL}, \end{array}$$ (7)

where *K* (= 4.87) is a constant and *α*_*BL*_ indicates the concentration of RBCs in the bottom layer, whose value was determined based on the conservation of RBC concentration between the WB and bottom layer in their study. Therefore, this approach has a limitation such that the value of constant *K* needs to be found for each condition. Furthermore, unlike the present model, the effect of particle interactions was not been explicitly included in this model.

By substituting the Eqs 4 and 5 into the continuity equations (Eqs 1 and 2) while imposing *j* = 0 at *r* = *R*_*o*_ (due to the closed bottom wall of the tube, see Fig 2A), we obtain

$$\begin{array}{c} \frac{\partial \alpha }{\partial t}+\frac{\partial }{\partial r}(r\frac{\omega^{2}}{g}f_{bk}\left(\alpha \right))=0. \end{array}$$ (8)

This is further non-dimensionalized by introducing the dimensionless variables of r* = *r*/*R*_*o*_, $f_{bk}^{*}=f_{bk}/u_{\infty }$ and t* = *tω*^2^*u*_∞_/*g*. Now, the final dimensionless form of our governing equation for the particle concentration is expressed as:

$$\begin{array}{c} \frac{\partial \alpha }{\partial t^{*}}+\frac{\partial }{\partial r^{*}}(r^{*}f_{bk}^{*}\left(\alpha \right))=0, \end{array}$$ (9)

which is a first-order quasi-linear partial differential equation that describes the centrifugal particle sedimentation. Instead of using the method of characteristics [26, 30], we solve the governing Eq 9 numerically to determine the positions of the interfaces between supernatant/suspension (*I*_*n* − *s*_) and suspension/sediment (*I*_*s* − *d*_) (Fig 2A). Actually, the analytical solution for Eq 9 is not in a completely explicit form and requires numerical integration as well, which is furthermore known to be highly dependent on the range of initial condition (particle concentration) [34]. Thus, being free from any constraints, we decided to get the solutions numerically for a wide parameter (initial condition) range. As a result, after spinning for certain time duration, it is possible to distinguish the concentration zones such as a clear liquid (*α* = 0), the retarded settling zone (0 < *α* ≤ *α*_*max*_) and the packed bed (bottom layer) (*α* = *α*_*max*_). To numerically solve the Eq 9, on the other hand, we use the modified upwind finite difference method [35] and the Engquist-Osher scheme [36] for extrapolating the flux density function. The details of the applied numerical algorithms are explained in elsewhere [35]. For our simulation, total 200 grid points (Δ*r* = 0.005*R*_*o*_) are uniformly distributed along the *r*-direction and the time steps of Δt* = ***O***(10^−3^) are used, determined by the CFL stability condition.

**Fig 3:**
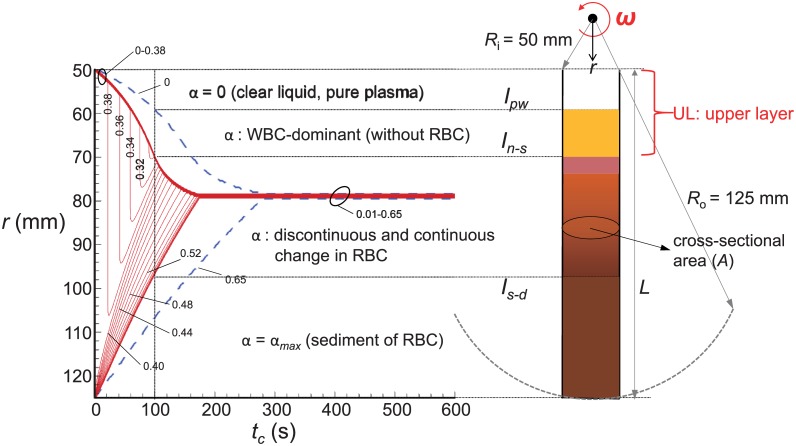
Temporal changes of RBC and WBC concentrations (*α*) in radial distribution inside the straight tube with centrifugal time (*t*_*c*_), predicted by one-dimensional unsteady particle sedimentation equation (Eq 9). Considered centrifugal acceleration is *a*_*c*_ = 1,000*g*, the maximum RBC concentration is *α*_*max*_ = 0.65, and the initial RBC concentration in the whole blood (i.e., Hematocrit) is *α*_*o*_ = 0.4. In the graph, the solid lines denote iso-concentration lines of RBC. Also, the iso-concentration lines of WBC under the same centrifugal and initial conditions as RBC are shown together (blue dashed lines) for comparison.

**Fig 4:**
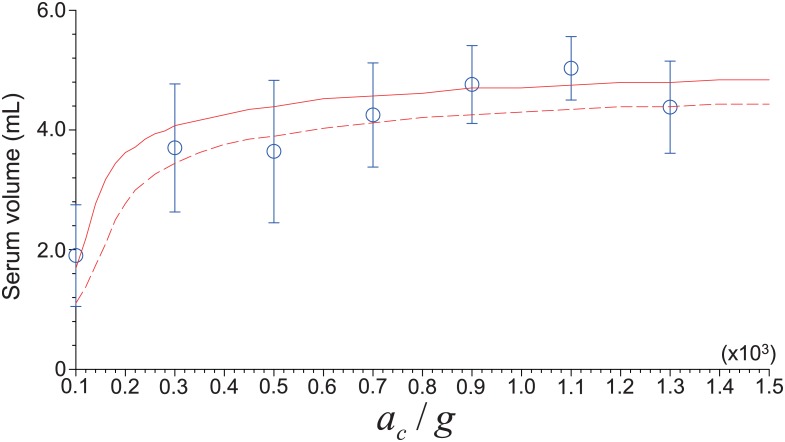
Comparison between the predicted and measured variations of volume of serum (*V*_*UL*_) with dimensionless centrifugal acceleration. ○, experimental data for the hematocrit range of 0.37–0.4 [16]. Lines (solid and dashed ones correspond to *H*_*e*_ = 0.37 and 0.4, respectively) denote the theoretical prediction based on *V*_*UL*_ = (*L* − *I*_*n* − *s*_)*A*. Centrifugation time and total blood volume are fixed at 10 minutes and 9 mL, respectively.

Fig 3 shows the typical distribution in the RBC concentration in a straight tube with the centrifugation time (*t*_*c*_), obtained by solving Eq 9, which is represented by the iso-concentration lines. Also, the WBC concentration distribution (see below for the method to predict it) under the same centrifugal condition is shown together. Here, it was assumed that the liquid-solid mixture is composed of plasma and RBCs (or WBCs) only (the maximum and initial concentrations of RBCs are chosen to be *α*_*max*_ = 0.65 and *α*_*o*_ = 0.4, respectively), and the applied centrifugal acceleration is fixed as *a*_*c*_ = 1,000*g*. The considered tube geometry, initially filled with the mixture of plasma and RBCs up to *L*, is also shown together in Fig 3. As we have explained above, it is now possible to determine the interfaces between regimes with different RBC concentrations. For example, after the tube is spun for 100 seconds, three distinct interfaces are detected depending on the variation in RBC concentration (Fig 3). In particular, for the case of monodisperse biosuspension like the present plasma-RBCs mixture in a tube, an upper layer (so-called PRP) consisting mostly of plasma can be determined in terms of the interface (*I*_*n* − *s*_) between the region without any RBCs (pure plasma) and that with RBCs. Thus, the recovery rate of the plasma (*E*_*plas*_) is calculated by [14, 21]:

$$\begin{array}{c} E_{plas}=\frac{V_{UL}}{V_{WB}\left(1-H_{e}\right)}, \end{array}$$ (10)

where *V*_*UL*_(= (*L* − *I*_*n* − *s*_)*A*) is the volume of the upper layer (*A*: cross-sectional area of tube (= *πD*^2^/4) and *L*: height of mixture), and *V*_*WB*_ and *H*_*e*_ denote the volume of whole blood and hematocrit, respectively. While the Eq 10 was derived from the reasoning that RBCs take up about 99% of the total cells in WB [21], it is also clear that the upper layer, typically called as a PRP in practical centrifugal separations, contains platelets as well as plasma. This is because the platelets, whose nominal size and density is about 1.0 *μm* and 1,050 *kg*/*m*^3^, which are much smaller than those (8.0 *μm* and 1,125 *kg*/*m*^3^) of RBC, would not migrate further to the tube bottom. Therefore, it would be possible to draw the relation between the recovery rate of platelets (*E*_*PLT*_) and that of plasma (*E*_*plas*_), which will be discussed in the next section. In the above formulation, we have approximated the blood cell as a rigid sphere to calculate the settling velocity (*u*_∞_) of a particle (Stokes’ law). As is well known, this assumption has been used widely by previous studies on the centrifugal cell separation (or elutriation) [21, 27, 29, 37–40]. In these studies, they modified the viscosity, density or the relative velocity of particles to compensate the uncertainties that may arise due to assuming blood cell as a sphere. For the same purpose, actually, we have used the modified settling velocity model by using the flux density function [32] (Eq 6). Due to the difficulty in the direct evaluation the assumption, instead we compare the volume of serum (i.e., the upper layer in Fig 3), *V*_*UL*_ = (*L* − *I*_*n* − *s*_)*A*, that is used to determine the recovery rate of plasma (Eq 10), obtained from experiment [16] and our prediction for the hematocrit range of *H*_*e*_ = 0.37–0.4. As shown in Fig 4, the *V*_*UL*_’s both from experiment and prediction show a good agreement each other, indicating that the assumption spherical particle is reasonable. On the other hand, it was reported that the density of red blood cell (*ρ*_*RBC*_) tends to vary during *in vivo* aging [41]. However, we have confirmed that the position of interface betweeen phases (*I*_*n* − *s*_) changes only about 1% with the reported range of density variation.

For the estimation of the recovery of white blood cells (WBCs), on the other hand, we start from the assumption that the particle-particle (i.e., RBCs-WBCs) interaction would be weak during the centrifugation, since the amount of WBCs is much smaller than that of RBCs. Then, the interface between pure plasma and WBC-dominant layer (*I*_*pW*_), and that between WBC-dominant and RBC-dominant layers (*I*_*n* − *s*_) is also determined by solving the governing equations (Eq 9) for WBCs and RBCs, separately. The exemplary locations of both interfaces that can be detected from the present approach are shown in Fig 3. It is again noted that the actual functionality of having WBCs in PRP is not the issue of present study. As a result, the recovery rate of WBC (*E*_*WBC*_) is simply obtained by

$$\begin{array}{c} E_{WBC}=c_{w}\frac{(I_{n-s}-I_{pW})A}{\beta_{o}V_{WB}}, \end{array}$$ (11)

where *c*_*w*_ is an empirical coefficient with an order magnitude of ***O***(10^−3^–10^−2^), introduced from previous studies of Sartory (1977) [42] and Berres *et al*. (2003) [43], and *β*_*o*_ (= 0.01) is the typical initial volume fraction of WBC in WB [44].

### Correlation model for platelet recovery rate

As we have explained in previous section, it is necessary and possible to draw a correlation between the recovery rates of platelets and plasma. Indeed, Brown (1989) [38] has claimed that the recovery rate of platelets is linearly proportional to that of plasma during centrifugal cell separation, i.e., *E*_*PLT*_/*E*_*plas*_ = constant (≃ 1.0). Based on the theory of scales of measurement [45, 46], on the other hand, the quantity *E*_*plas*_ and *E*_*PLT*_ are classified as the ratio scale-types, and the admissible relation between the independent quantity *E*_*plas*_ and dependent quantity *E*_*PLT*_ can be expressed as a product form of

$$\begin{array}{c} E_{PLT}{\left(E_{plas}\right)}^{a}=b. \end{array}$$ (12)

**Fig 5:**
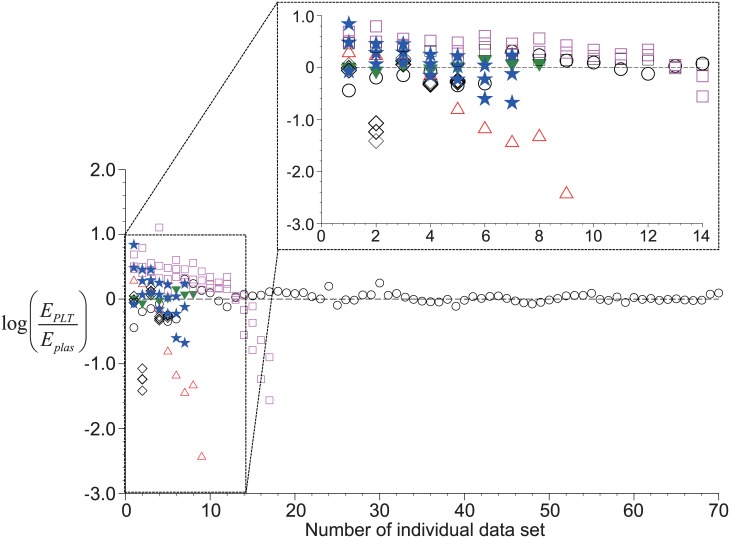
Distribution of the ratio of platelet recovery rate (*E*_*PLT*_) to that of plasma (*E*_*plas*_) from the experimental data available in the literature. ▼, Kahn *et al*. (1976) [14]; ○, Brown (1989) [38]; ◻, Araki *et al*. (2012) [15]; △, Perez *et al*. (2013) [21]; ★, Jo *et al*. (2013) [16]; ◊, Amable *et al*. (2013) [23].

For conveniences, logarithmic form of Eq 12, *a* log(*E*_*plas*_) + log(*E*_*PLT*_) = log(*b*) is usually used, and *a* is a constant that is independent on any other quantities while *b* is dependent on other quantities. So the correlation model by Brown (1989) [38] corresponds to the case of *a* = −1 and *b* = 1 such that log(*E*_*PLT*_/*E*_*plas*_) = 0, indicating that the ratio of recovery rate of platelets to plasma is independent on any other variables. While gathering and analyzing the actual clinical data [14–16, 21, 23] available in the literature, however, we found that the above relation is not always valid. Fig 5 shows the scattering of actual values of log(*E*_*PLT*_/*E*_*plas*_) gathered from the above literatures together with that of Brown (1989) [38]. Considering the fact that the ranges of centrifugal time and acceleration, tube geometry, and the initial condition of tested blood are widely different between the referred studies, we think it is quite necessary to draw a more reliable (i.e., applicable to a more wide range of conditions) model to correlate the platelet recovery rate to that of plasma.

To achieve this, we first perform a dimensional analysis, i.e., Buckingham Pi-theorem [47, 48]. Based on our physical intuition and results from previous studies, it is reasonably deduced that the recovery rate of platelets (*E*_*PLT*_) is determined by various variables as shown:

$$\begin{array}{c} E_{PLT}=f\left(A,L,u_{\infty },U_{o},t_{c},V_{t},E_{plas}\right). \end{array}$$ (13)

Here, $u_{\infty }=\left(\rho_{s}-\rho_{l}\right)d_{s}^{2}g/18\mu_{l}$ and $U_{o}=\left(\rho_{s}-\rho_{l}\right)d_{s}^{2}\left(\omega^{2}R_{o}\right)/18\mu_{l}$ are the terminal (settling) velocity of a single particle under the gravitational and centrifugal accelerations, respectively, and *V*_*t*_ is the volume of the tube. The above functional form also indicates that log(*E*_*PLT*_/*E*_*plas*_) should be expressed as Eq 14, rather than being a constant of 0 as suggested by Brown (1989) [38].

$$\begin{array}{c} \text{log}(\frac{E_{PLT}}{E_{plas}})=f\left(A,L,u_{\infty },U_{o},t_{c},V_{t}\right). \end{array}$$ (14)

**Fig 6:**
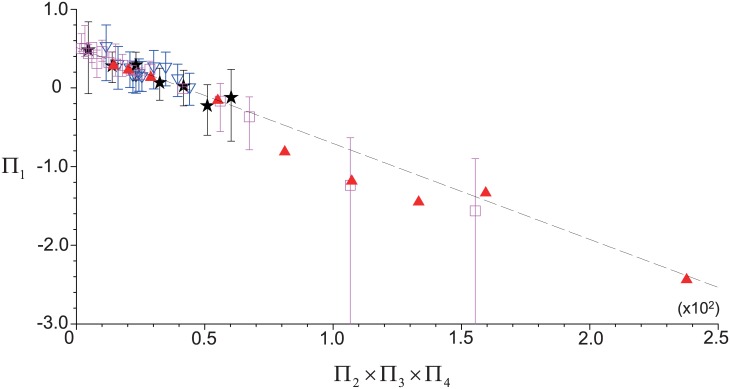
Variation of Π_1_ with Π_2_Π_3_Π_4_ for the available experimental data. ▲, from Perez *et al*. (2013) [21] (*V*_*WB*_ = 3.5 mL *t*_*c*_ = 10 minutes); ◻, Araki *et al*. (2012) [15] (*V*_*WB*_ = 7.5 mL, *t*_*c*_ = 10 minutes); ★, Jo *et al*. (2013) [16] (*V*_*WB*_ = 9.0 mL, *t*_*c*_ = 10 minutes); ▽, Jo *et al*. (2015) (*V*_*WB*_ = 9.0 mL, *t*_*c*_ = 5 minutes). - - -, linear regression function of Π_1_ = −0.0122Π_2_Π_3_Π_4_ + 0.5128.

Here, the centrifugal time (*t*_*c*_), cross-sectional area of the tube (*A*) and the initial volume of whole blood (*AL*) are considered as fundamental kinds of units [47] based on their physical importance in the relation. To constitute dimensionless groups (i.e., Π’*s*) with a physical dominance using Buckingham’s Pi-theorem, first of all, we need to classify the variables in Eq 14 into primary and repeating ones, respectively. Since the major goal of present model is to optimize the centrifugal parameters and initial conditions in PRP preparation, it is reasonable to consider *V*_*t*_, *U*_*o*_ and *t*_*c*_ as variables of interest, while taking *A*, *L* and *u*_∞_ as repeating variables. Thus, from Pi-theorem, we can derive four Π’s as:

$$\begin{array}{c} \Pi_{1}=\text{log}(\frac{E_{PLT}}{E_{plas}}),\Pi_{2}=\frac{V_{t}}{AL},\Pi_{3}=\frac{t_{c}}{L/u_{\infty }},\Pi_{4}=\frac{U_{o}}{u_{\infty }}=\frac{\omega^{2}R_{o}}{g}, \end{array}$$ (15)

where Π_1_ denotes the recovery rate ratio of platelets to plasma, Π_2_ is the volume fraction of WB relative to the tube capacity, Π_3_ shows the characteristic time scale, and Π_4_ explains the ratio of centrifugal acceleration to gravitational one (*g*), respectively. Consequently, a generalized correlation between Pi-groups based on a power function is expressed as:

$$\begin{array}{c} \Pi_{1}=c_{1}{\left(\Pi_{2}\right)}^{e_{2}}{\left(\Pi_{3}\right)}^{e_{3}}{\left(\Pi_{4}\right)}^{e_{4}}+c_{2}, \end{array}$$ (16)

where the coefficients (*c*_1_ and *c*_2_) and exponents (*e*_2_, *e*_3_ and *e*_4_) are determined through the regression method with existing experimental data. In the present study, we used the experimental data of Araki *et al*. (2012) [15], Jo *et al*. (2013) [16], and Perez *et al*. (2013) [21], in which all the information necessary to complete the above relation was available (the validation will be discussed later). That is, as shown in Fig 6, we plotted the variations of Π_1_ with Π_2_Π_3_Π_4_ for the available experimental data from different conditions and they collpse into a single linear function of Π_1_ = −0.0122Π_2_Π_3_Π_4_ + 0.5128. As a result, the equation between Pi-groups is established as:

$$\begin{array}{c} \Pi_{1}=-0.0122\Pi_{2}\Pi_{3}\Pi_{4}+0.5128. \end{array}$$ (17)

As shown, all the exponents are 1.0, which indicates that the logarithmic ratio of *E*_*PLT*_ to *E*_*plas*_ has a linear relationship with the dimensionless variables (Π_2_, Π_3_ and Π_4_) when any two of them is fixed. Finally, the correlation model between *E*_*PLT*_ and *E*_*plas*_ is obtained as:

$$\begin{array}{c} \frac{E_{PLT}}{E_{plas}}=1.67\text{exp}(-0.0122\frac{V_{t}}{AL}\cdot \frac{t_{c}}{L/u_{\infty }}\cdot \frac{\omega^{2}R_{o}}{g}). \end{array}$$ (18)

As noted, this correlation describes *E*_*PLT*_/*E*_*plas*_ as a function of the whole blood volume, centrifugal time and acceleration, with which the platelet recovery rate (*E*_*PLT*_) can be determined once the recovery of plasma (*E*_*plas*_) is obtained by solving the Eq 9.

**Fig 7:**
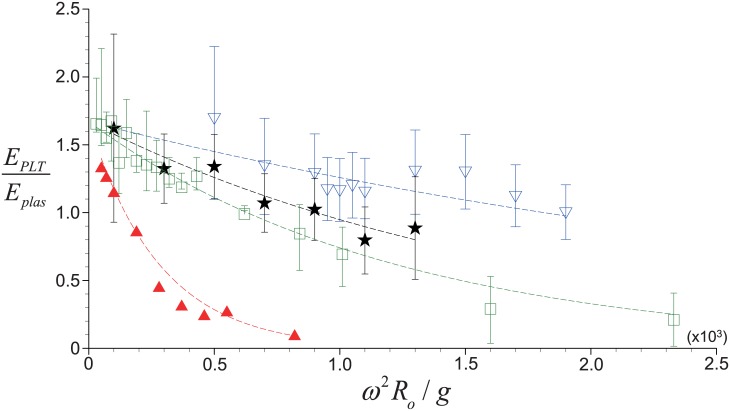
Comparison between the predicted and measured variations of *E*_*PLT*_/*E*_*plas*_ with dimensionless centrifugal acceleration. —, theoretical prediction based on present model; △, experimental data from Perez *et al*. (2013) [21] (*V*_*WB*_ = 3.5 mL *t*_*c*_ = 10 minutes); ◻, Araki *et al*. (2012) [15] (*V*_*WB*_ = 7.5 mL, *t*_*c*_ = 10 minutes); ★, Jo *et al*. (2013) [16] (*V*_*WB*_ = 9.0 mL, *t*_*c*_ = 10 minutes); ▽, Jo *et al*. (2013) [16] (*V*_*WB*_ = 9.0 mL, *t*_*c*_ = 5 minutes).

To further discuss the reliability of our model, we have compared the values of *E*_*PLT*_/*E*_*plas*_ measured under different conditions [15, 16, 21] in Fig 7 with our predictions where each clinical environment has been fully incorporated. It is found that for each data set, the recovery rate ratio shows an exponential decay with increasing centrifugal acceleration, which agrees well with our model. Furthermore, the decaying rate strongly depends on the centrifugal time (*t*_*c*_) and whole blood volume (*V*_*WB*_). As the centrifugation time increases while fixing *V*_*WB*_, for example, the decaying rate becomes much faster; however, the increase of *V*_*WB*_ (with a fixed *t*_*c*_) would reduce the rate. This shows that we may obtain a high *E*_*PLT*_/*E*_*plas*_ with a short centrifugation time or large blood volume with the same centrifugal acceleration, and there should be an optimal combination of controlled parameters to maximize the recovery rate of platelets. Later, we will discuss this condition in detail. Finally, we would like to emphasize that it was not possible to capture all these features with the previous correlation model by Brown (1989) [38], but our new model can successfully explain the physical details involved in the centrifugal separation of blood cells.

### Determination of the parameter ranges

To cover various conditions in practice, a range of parameters such as volume of whole blood (WB), tube geometry (e.g., cross-sectional area), hematocrit (volume fraction of RBCs in WB) and centrifugal condition (centrifugal time and acceleration) are considered in the present study. The initial volume of WB is varied as 3.5, 7.0 and 9.0 mL, based on the actual experiments by Perez *et al*. (2013) [21], Araki *et al*. (2012) [15], and Jo *et al*. (2013) [16], respectively, while the typical tube geometry such as *A* = 120 *mm*^2^ and *V*_*t*_ = 15 mL is considered. The distance between the center of rotation and tube bottom is *R*_*o*_ = 150 *mm*. To investigate the effect of tube geometry, we additionally consider the conical bottom shape as well (Fig 2B). We vary the hematocrit in the range of *H*_*e*_ = 0.37–0.52, reflecting the difference between individuals (e.g., difference between male and female) and also the maximum concentration of RBCs due to packing at the tube bottom is limited by defining *α*_*max*_ = 0.80, caused by the deformation of the blood cells [27, 29, 33]. This *α*_*max*_ is defined such that the flux through the interface bordering the packed bed saturates to zero and for biosuspensions (such as RBCs) it has been set to be 0.8 [27, 33, 49]. Finally the ranges of centrifugal time and acceleration are determined based on the typical values used in clinical practices; so that the centrifugal time is varied as *t*_*c*_ = 2–15 minutes and the centrifugal acceleration covers the range of *a*_*c*_ = 100*g*–1,500*g* (*g* = 9.81 *m*/*s*^2^) [14–16, 21–24, 50].

In predicting recovery rates of cells during centrifugation, in addition, we consider realistic properties of blood cells. For example, the size and density for RBC, WBC, and platelet are set to be *d*_*RBC*_ = 8 *μm*, *ρ*_*RBC*_ = 1,125 *kg*/*m*^3^; *d*_*WBC*_ = 10 *μm*, *ρ*_*WBC*_ = 1,065 *kg*/*m*^3^; and *d*_*PLT*_ = 1 *μm*, *ρ*_*PLT*_ = 1,050 *kg*/*m*^3^, respectively. For plasma, the density and viscosity are used as *ρ*_*plas*_ = 1,032 *kg*/*m*^3^ and *μ*_*plas*_ = 1.0 *mPa* ⋅ *s*, respectively [38, 51].

## Results and discussion

### Comparison between prediction and experimental data: Validation

**Fig 8:**
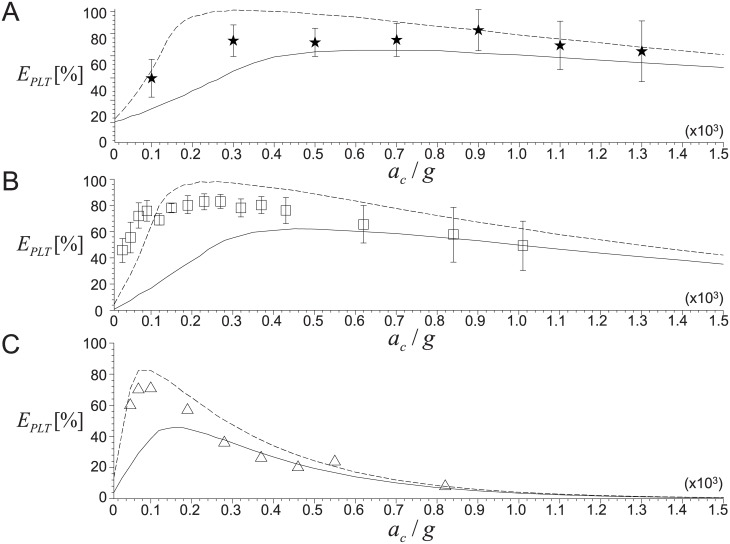
Variations of platelet recovery rate (*E*_*PLT*_) with centrifugal acceleration (*a*_*c*_ = *ω*^2^*R*_*o*_). A: *V*_*WB*_ = 9.0 mL (★, from Jo *et al*. (2013) [16]); B: 7.5 mL (◻, from Araki *et al*. (2012) [15]); C: 3.5 mL (△, from Perez *et al*. (2013) [21]). In the figure, lines denote the present theoretical predictions (- - -, *H*_*e*_ = 0.37; —, 0.52). Centrifugal time is fixed as *t*_*c*_ = 10 minutes.

In this section, we will compare the predicted recovery rates of platelets and WBCs with the experimental data available in the literature. Fig 8 shows the variation of the recovery rates of platelets (*E*_*PLT*_) with centrifugal acceleration (*a*_*c*_ = *ω*^2^*R*_*o*_) for three cases of *V*_*WB*_ = 3.5, 7.0 and 9.0 mL, while the centrifugal time is fixed as *t*_*c*_ = 10 minutes. Here, the compared experimental data were adopted from three different studies [15, 16, 21], indicating that they were obtained under different environments that were reflected in the prediction with the present model. Although almost all information to run our model were available, the exact value of hematocrit (*H*_*e*_) was not specified and thus we used its typical range of *H*_*e*_ = 0.37–0.52, of which the boundary is shown as dashed and solid lines in each plot in Fig 8. It is clearly shown that the present model predicts the dependency of *E*_*PLT*_ on the centrifugal acceleration pretty well; in particular, the critical centrifugal acceleration (i.e., the spinning speed of centrifugation) for the maximum *E*_*PLT*_ matches well with the experimental data. As a smaller volume of whole blood is used, the maximum *E*_*PLT*_ achievable from the centrifugation is reduced, while the critical centrifugal acceleration decreases as well. In the following sections, we will discuss the effects of these principal variables in detail. On the other hand, the predicted *E*_*PLT*_ shows a slight deviation from the experimental data for the centrifugal accelerations smaller than about 100*g* (see Fig 8B, for example). This is because the contribution of the gravitational acceleration would not be negligible as the centrifugal acceleration becomes smaller [26, 30]. In formulating our model, we ignored the effect of gravitational force that acts along the direction perpendicular to the sedimentation direction (see Fig 2A).

**Fig 9:**
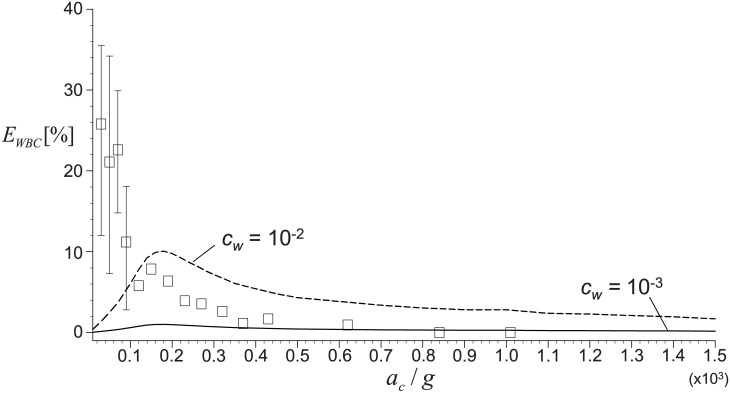
Variation of white blood cell recovery rate (*E*_*WBC*_) with centrifugal acceleration (*a*_*c*_). Centrifugal time fixed as *t*_*c*_ = 10 minutes and *V*_*WB*_ is 7.5 mL. Solid lines denote the boundaries of the predicted *E*_*WBC*_ for the range of *c*_*w*_ = 10^−3^–10^−2^, and ◻’s are from Araki *et al*. (2012) [15].

In Fig 9, we also validated the accuracy of predicted recovery rates of WBCs (Eq 11). Unlike the case of platelet recovery, less experimental data were available for the inclusion of WBC’s in PRP and single data set from Araki *et al*. (2012) [15] was compared in the present study. This is because the separation and recovery of WBCs during PRP preparation has not been paid attention much due to the controversy about the clinical role of WBCs in PRP [9]. In the figure, the solid lines denote the boundaries of the predicted *E*_*WBC*_ for the range of coefficient *c*_*w*_ (10^−3^–10^−2^) [42, 43]. It is found that our prediction captures the same trend of varying *E*_*WBC*_ according to the centrifugal acceleration; in particular, the critical acceleration to achieve the maximum recovery of WBCs agrees well with the experimental data. Similar to the platelet recovery, the deviation between our prediction and experiment becomes larger at smaller centrifugal acceleration (< 100*g*). To our best knowledge, this is the first attempt to predict the recovery rate of WBCs in PRP preparation and the results are quite promising.

### Effects of centrifugal conditions on the recovery of blood cells in PRP

**Fig 10:**
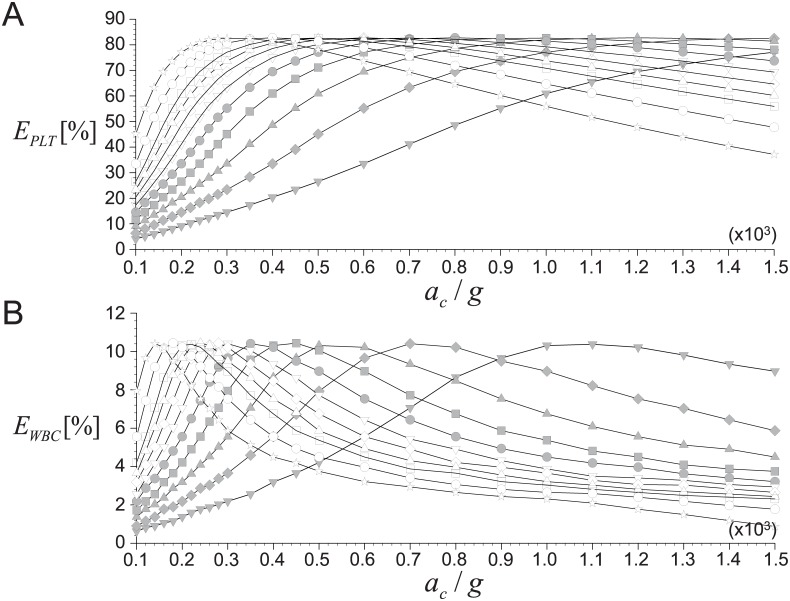
Effects of centrifugal time (*t*_*c*_) on the recovery rates. A: *E*_*PLT*_; B: *E*_*WBC*_. ▼, *t*_*c*_ = 2 minutes; ♦, 3 minutes; ▲, 4 minutes; ■, 5 minutes; ⚫, 6 minutes; ▽, 7 minutes; ◊, 8 minutes; △, 9 minutes; ◻, 10 minutes; ○, 12 minutes; ⋆, 15 minutes. *V*_*WB*_ is fixed as 9 mL.

Now, we discuss the effects of centrifugal conditions (i.e., time *t*_*c*_ and acceleration *a*_*c*_) on the recovery rates of platelets (*E*_*PLT*_) and WBCs (*E*_*WBC*_) based on the theoretical predictions developed in the present study. Shown in Fig 10 are the variations of the averaged (for the ranges of *H*_*e*_ = 0.37–0.52 and *c*_*w*_ = 10^−3^–10^−2^, respectively) recovery rates of platelets and WBCs with varying *t*_*c*_ and *a*_*c*_. The maximum concentration of blood cells is set as *α*_*max*_ = 0.8 and *V*_*WB*_ = 9 mL is considered. When the spinning speed increases with a fixed time, both *E*_*PLT*_ and *E*_*WBC*_ increase sharply to approach the maximum value and then decreases relatively slowly. This dependency on centrifugal acceleration can be also found in the recent experimental studies [15, 16, 24], but they considered a single value of *t*_*c*_ while our predictions make it possible to perform a parametric study. As shown in Fig 10A, when *t*_*c*_ is as small as 2 minutes, *E*_*PLT*_ increases slowly and does not reach the maximum even with the acceleration as large as *a*_*c*_ = 1,500*g*. This is because the centrifugal time is not long enough to separate the plasma and RBCs successfully. As *t*_*c*_ increases, the slope of increasing *E*_*PLT*_ becomes steeper and a larger recovery of platelets (over 80%) is possible at a critical *a*_*c*_ that tends to get smaller as the centrifugation is performed longer. This information would provide us a good strategy to achieve the maximum platelet recovery by optimizing the condition for practical application. For example, the critical *a*_*c*_ becomes as small as about 350*g* at *t*_*c*_ = 12 minutes, which will be very helpful in maintaining the platelet integrity [14, 22, 24] as well as maximizing the platelet recovery. Interestingly, the maximum recovery rate of platelet achievable at each critical *a*_*c*_ does not change much with varying *t*_*c*_, which may indicate that the maximum *E*_*PLT*_ depends on the other conditions such as hematocrit, whole blood volume and tube geometry rather than the centrifugal time and acceleration (details will be discussed below).

Fig 10B shows the effects of centrifugal conditions (*t*_*c*_ and *a*_*c*_) on the recovery rate of WBCs (*E*_*WBC*_). The general trends of *E*_*WBC*_ with *t*_*c*_ and *a*_*c*_ are similar to those of *E*_*PLT*_; that is, with increasing *a*_*c*_, *E*_*WBC*_ increases to the maximum at a critical *a*_*c*_, and then decreases back. The slope of *E*_*WBC*_ change with *a*_*c*_ and the critical *a*_*c*_ show similar variations with those of *E*_*PLT*_, and the maximum *E*_*WBC*_ is maintained constant despite varying centrifugal conditions. On the other hand, it is noted that the critical *a*_*c*_ to achieve the maximum *E*_*WBC*_ is smaller than that for the maximum *E*_*PLT*_, which agrees with the experimental data of Araki *et al*. (2012) [15]. This is because the time (velocity) scale of the platelets is much smaller than that of WBCs due to the large difference in their sizes and densities. Again, we think this is one of advantages of our theoretical model to capture this difference accurately.

**Fig 11:**
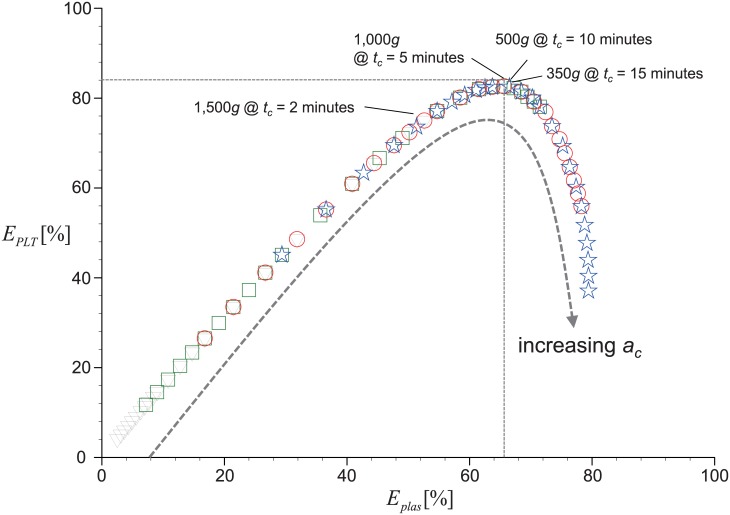
Variation of *E*_*PLT*_ with *E*_*plas*_ for the range of *a*_*c*_ = 100*g* – 1,500*g* at selected *t*_*c*_. ▽, 2 minutes; ◻, 5 minutes; ○, 10 minutes; ⋆, 15 minutes. *V*_*WB*_ is fixed as 9 mL.

After the feasibility of our model is confirmed, we will discuss more on the details of our model. First of all, to understand this discrepancy between the present model and Brown’s assumption [38], it would be meaningful to collect the estimated recovery rates of platelets and plasma as a polar plot, as shown in Fig 11. The data set shown in the figure includes the cases of *t*_*c*_ = 2, 5, 10, and 15 minutes with *a*_*c*_ = 100*g*–1,500*g*. It is interesting to see that all the collected data collapse into a single parabolic curve. As we have introduced above, Brown (1989) [38] has proposed that the polar plot between *E*_*PLT*_ and *E*_*plas*_ has a linear correlation, which actually corresponds to the regime before the maximum *E*_*PLT*_ is achieved (Fig 11). That is, in our analysis, *E*_*PLT*_ is found to increase almost linearly with increasing *E*_*plas*_ until it reaches the maximum. Thus, we may imagine that the Brown’s result has been established based on the incomplete data set. After the maximum *E*_*PLT*_ is achieved, it begins to decrease with further increase of *a*_*c*_; on the other hand, *E*_*plas*_ steadily increases. In spite of different centrifugation times, the collapse of the data into a single curve indicates that the maximum *E*_*PLT*_ and the corresponding *E*_*plas*_ are maintained the same. This suggests that there exists a critical *E*_*plas*_, determined by the volume of the upper layer, so-called PRP, in which the amount of recovered platelets is maximized. It is again shown that *E*_*plas*_ increases with increasing *t*_*c*_, which would reach the critical *E*_*plas*_ at lower *a*_*c*_, indicating that the critical *a*_*c*_ for the maximum *E*_*PLT*_ decreases with increasing *t*_*c*_.

### Effects of whole blood volume, hematocrit and tube geometry

**Fig 12:**
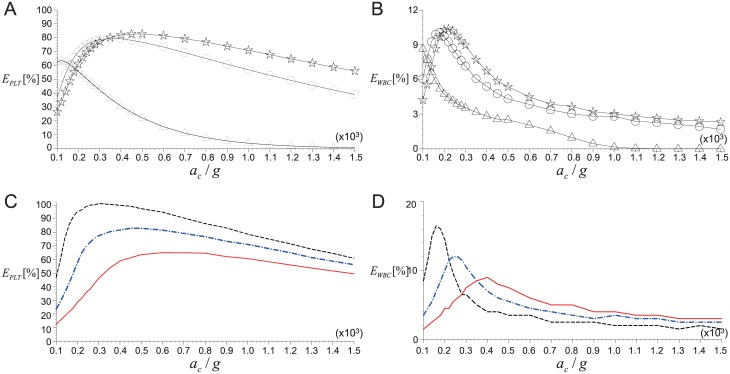
Effects of whole blood volume and hematocrit. A: *V*_*WB*_ on *E*_*PLT*_; B: *V*_*WB*_ on *E*_*WBC*_; C: *H*_*e*_ on *E*_*PLT*_; D: *H*_*e*_ on *E*_*WBC*_. In (A) and (B), ⋆, *V*_*WB*_ = 9 mL; ○, 7.5 mL; △, 3.5 mL. In (C) and (D), - - -, *H*_*e*_ = 0.37; − ⋅ − ⋅ −, 0.45; ——, 0.52. Centrifugation time is fixed as *t*_*c*_ = 10 minutes.

The recovery rate of blood cells would be also strongly influenced by the conditions such as the volume of whole blood, hematocrit, and tube geometry. Fig 12 shows the variations of *E*_*PLT*_ and *E*_*WBC*_ with *a*_*c*_ (*t*_*c*_ is fixed as 10 minutes) while varying the volume of whole blood as *V*_*WB*_ = 3.5–9.0 mL and hematocrit as *H*_*e*_ = 0.37–0.52. First of all, the effect of *V*_*WB*_ on the averaged (for the range of *H*_*e*_ = 0.37–0.52) *E*_*PLT*_ and *E*_*WBC*_ is plotted in Fig 12A and 12B. When *V*_*WB*_ is as small as 3.5 mL, *E*_*PLT*_ reaches the maximum (∼63%) at a very low centrifugal acceleration of 120*g* that is close to the optimal *a*_*c*_ of 100*g* measured by Perez *et al*. (2013) [21] who used the same *V*_*WB*_. On the whole, with increasing *V*_*WB*_, the maximum achievable *E*_*PLT*_ and corresponding critical *a*_*c*_ tend to increase (Fig 12A). Thus, even considering the limited capacity of a tube (typically 15 mL in real applications), it is recommended to collect more blood to maximize the recovery rate of platelets. The recovery of WBCs shows a similar trend such that the maximum *E*_*WBC*_ (and critical *a*_*c*_) increases as *V*_*WB*_ increases (Fig 12B). As we have shown in Fig 10, the magnitude of *E*_*PLT*_ is usually higher than *E*_*WBC*_, which has been measured in previous experimental studies [15, 21], as well. This is because not only do WBCs have a very small (< 0.01) initial volume fraction, but they also exist in a thin intermediate layer in PRP such that it is easy for them to be lost into the packing bed of RBCs. Finally, it should be again noted that the critical *a*_*c*_ for the maximum *E*_*WBC*_ is not the same as the one for the maximum *E*_*PLT*_, which is useful to choose the most efficient set of centrifugal conditions at a given clinical condition.

Shown in Fig 12C are the variations of *E*_*PLT*_ with *a*_*c*_ for three difference cases of hematocrit as *H*_*e*_ = 0.37, 0.45 and 0.52. Again, these values of hematocrit were chosen to reflect the variations in real human data [52]. Other parameters are fixed as *V*_*WB*_ = 9 mL and *t*_*c*_ = 10 minutes. As *H*_*e*_ increases, the maximum *E*_*PLT*_ decreases significantly while the corresponding critical *a*_*c*_ increases. In this sense, it would be helpful to have a smaller volume fraction of RBCs in the whole blood to have more platelets in PRP, which explains the reason why previous studies have diluted the blood before centrifugation [17, 21]. Similarly, the maximum *E*_*WBC*_ and the corresponding critical *a*_*c*_ are predicted to increase and decrease, respectively, as the initial volume fraction of RBCs decreases (Fig 12D). As shown, the change rate of *E*_*WBC*_ with *a*_*c*_ near the maximum peak becomes much steeper as *H*_*e*_ decreases, indicating that the *E*_*WBC*_ is more sensitive to *a*_*c*_ at lower *H*_*e*_. Therefore, we may imagine that controlling the separation and recovery of WBCs in PRP would be very difficult in the actual clinical environment.

**Fig 13:**
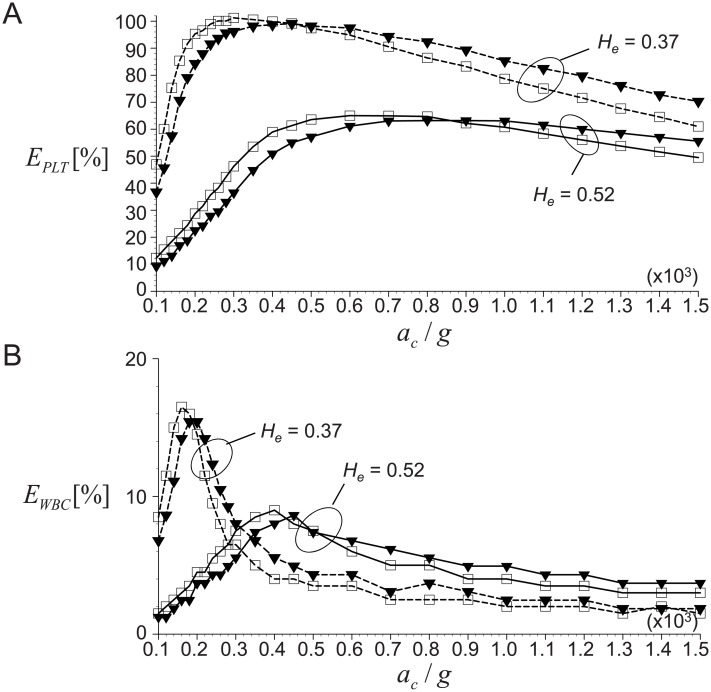
Effects of tube bottom geometry on the recovery rates. A: Platelet; B: WBC. ◻, flat type; ▼, conical type. Centrifugal time is fixed as *t*_*c*_ = 10 minutes and *V*_*WB*_ is 9 mL.

Finally, to compare the effect of bottom shape of tube (flat bottom as a reference), we have additionally tested the conical bottom shape, which has been adopted from the shape of *BD Falcon conical tube* that is widely used in the actual clinical test. Typically, the height of conical part is about one fifth of the total length and the diameter of upper base has one quarter of the diameter of the tube. In the present study, the conical shape with the height of 0.2*R*_*o*_ (*R*_*o*_, distance between the center of rotation and tube bottom) and the diameter of upper base of 0.25*D* (*D*, width of the tube) has been considered (see Fig 2B). Fig 13 shows the effect of tube geometry (i.e., flat and conical shapes at the bottom) on *E*_*PLT*_ and *E*_*WBC*_, while we vary *H*_*e*_ as 0.37 and 0.52 with *V*_*WB*_ = 9.0 mL and *t*_*c*_ = 10 minutes. In general, the overall dependency of *E*_*PLT*_ and *E*_*WBC*_ on *a*_*c*_ is not affected much by varying the cross-sectional area of tube bottom; however, the maximum achievable *E*_*PLT*_ and *E*_*WBC*_ (and corresponding critical *a*_*c*_) have changed. Compared to the flat type, the conical type produces smaller maximum recovery of blood cells and the corresponding critical centrifugal acceleration becomes larger. For example, for the flat bottom geometry, *E*_*PLT*_ reaches the maximum (∼100%) at *a*_*c*_ = 300*g* with *H*_*e*_ = 0.37, but that of the conical tube has the maximum (∼98%) at *a*_*c*_ = 450*g* (Fig 13A). As *a*_*c*_ increases toward the critical value, the predicted *E*_*PLT*_ (and *E*_*WBC*_) in the flat type is higher than that in the conical type. Interestingly, after the maximum recovery is achieved, this phenomenon is reversed at *a*_*c*_ between 800*g* and 900*g*. To understand the reasons for this (especially focusing on *E*_*PLT*_), we perform a scaling analysis based on the Eqs 10 and 18 that were used to determine the recovery rate of platelets. As shown below, it is possible to draw simple scaling relations for the change rates of *E*_*plas*_ and *E*_*PLT*_/*E*_*plas*_ on *a*_*c*_.

$$\begin{array}{c} \frac{\partial }{\partial a_{c}}(E_{plas})∼\frac{u_{\infty }t_{c}}{L(1-H_{e})},\frac{\partial }{\partial a_{c}}(\frac{E_{PLT}}{E_{plas}})∼-\frac{V_{t}u_{\infty }t_{c}}{AL^{2}}\text{exp}(-\frac{V_{t}u_{\infty }t_{c}}{AL^{2}}a_{c}). \end{array}$$ (19)

**Fig 14:**
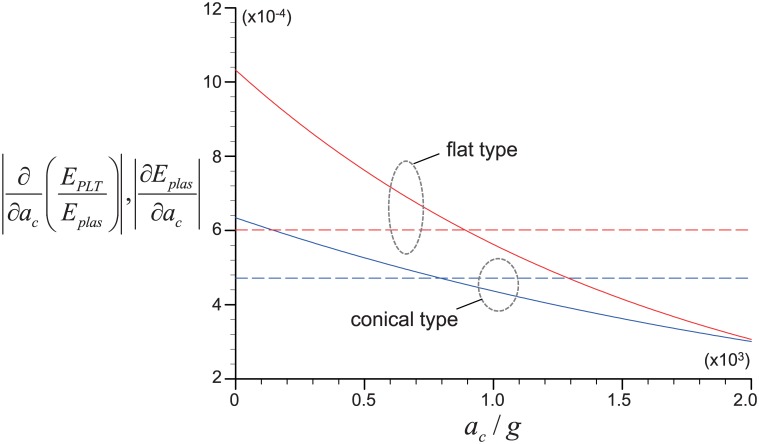
Variation of ∂(*E*_*PLT*_/*E*_*plas*_)/∂*a*_*c*_ (solid lines) and ∂(*E*_*plas*_)/∂*a*_*c*_ (dashed lines) with *a*_*c*_ for flat and conical bottom shapes. Considered parameters are same as those used for the case of *H*_*e*_ = 0.52 in Fig 13A.

Once all variables for centrifugal separation of blood cells are pre-determined, the first relation shows that ∂(*E*_*plas*_)/∂*a*_*c*_ is a positive constant indicating the gathering rate of platelets in the PRP. On the other hand, the second relation, a decreasing function of *a*_*c*_ in negative values, denotes the rate of platelet loss to the packed bed regime. Thus, in Fig 14, we have plotted the variations of above two quantities (in absolute values) for the cases of *H*_*e*_ = 0.52 shown in Fig 13A. Here, we can find a few interesting things. First of all, for both bottom shapes, it is shown that the two ratios cross each other at *a*_*c*_ = 800*g*–900*g* (= *a*_*cr*_) which is close to the centrifugal acceleration where *E*_*PLT*_’s from both cases are reversed (see Fig 13A). So, it is understood that the loss of *E*_*PLT*_ would be faster at *a*_*c*_ < *a*_*cr*_ while more platelets will be gathered at *a*_*c*_ > *a*_*cr*_. Second, the change rates of both *E*_*plas*_ and *E*_*PLT*_/*E*_*plas*_ with *a*_*c*_ are higher for the flat type, compared to the conical one. Therefore, as shown in Fig 13A, the platelet recovery rate on flat type will be higher before the above two rates are balanced (i.e., *a*_*c*_ < *a*_*cr*_) while the conical one will prevail at *a*_*c*_ > *a*_*cr*_.

### Optimization of centrifugal conditions for PRP preparation

**Fig 15:**
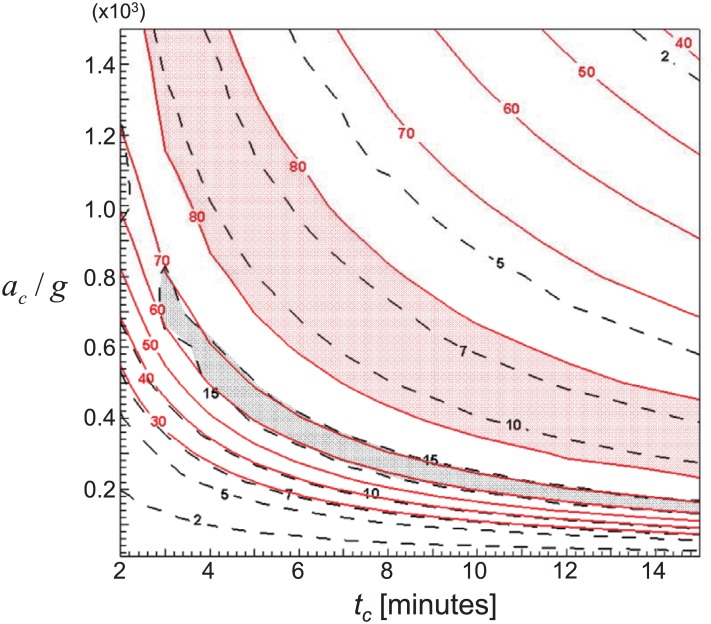
Contours of *E*_*PLT*_ (solid lines) and *E*_*WBC*_ (dashed lines) with centrifugal time (*t*_*c*_) and acceleration (*a*_*c*_). *V*_*WB*_ is fixed as 9.0 mL.

In previous sections, the effects of centrifugal conditions, whole blood volume, hematocrit, and tube geometry on recovery rates of platelets and WBCs have been discussed. Among these parameters, it is most frequently required to optimize the centrifugal conditions in real clinical applications. By mapping the predicted *E*_*PLT*_ and *E*_*WBC*_ together, it is possible to provide a simple guideline to optimize them. For example, Fig 15 shows the contours of averaged (for the range of *H*_*e*_ = 0.37–0.52) *E*_*PLT*_ and *E*_*WBC*_ with *a*_*c*_ and *t*_*c*_ (whole blood volume is fixed as 9 mL and the flat tube bottom geometry was considered). It is clearly observed that there are two regions for large *E*_*WBC*_ (≥ ∼ 15%) and *E*_*PLT*_ (≥ ∼ 80%), and depending on the target it is feasible to select the appropriate centrifugal conditions. If one wishes to achieve the time efficiency, about 82% *E*_*PLT*_ and 10% *E*_*WBC*_ would be obtained at the centrifugal acceleration of 1,500*g* for a relatively short spinning time of *t*_*c*_ = 2.5 minutes, or about 70% *E*_*PLT*_ and 15% *E*_*WBC*_ are obtained at *a*_*c*_ = 1,200*g* and *t*_*c*_ = 2.0 minutes. On the other hand, to achieve the enhanced platelet integrity, it is typically recommended to apply smaller centrifugal acceleration, which will require longer centrifugation time to maximize the recovery rates. For example, about 81% *E*_*PLT*_ and 11% *E*_*WBC*_ are achieved at *a*_*c*_ = 240*g* for *t*_*c*_ = 15 minutes, or about 63% *E*_*PLT*_ and 16% *E*_*WBC*_ are at *a*_*c*_ = 140*g* for *t*_*c*_ = 15 minutes. In general, it is understood that the trade-off between the time efficiency and platelet integrity should be considered in practical applications. In this case, the optimal set of centrifugal conditions consisting of the moderate centrifugal acceleration between 700*g*–800*g* and relatively short spinning time (4–5 minutes) would be selected to obtain 80% *E*_*PLT*_ and 12% *E*_*WBC*_. Once the important environments are fully considered in the prediction, such as tube geometry, hematocrit and volume of whole blood, that may cause the large discrepancy between the optimal centrifugal conditions reported from previous studies, it is possible to generate this kind of map to select the spinning time and speed according to the specific cases. We would like to finally emphasize that this optimization was not possible in previous attempts.

## Conclusion

In the present study, challenged by the large discrepancy between the recently proposed optimal centrifugal conditions for platelet-rich plasma (PRP) preparation, we developed a theoretical model, based on the knowledge in multiphase flow, to predict the recovery rates of the platelets and white blood cells in the process of centrifugal separation of the whole blood in a tube for the preparation of PRP. For a range of practical parameters, such as whole blood volume, hematocrit and centrifugal conditions (i.e., time and acceleration), the predicted recovery rates show good agreements with available experimental data. The dependence of the recovery rate (platelets and WBCs) on centrifugal conditions is found such that there exist the critical time and acceleration to achieve the maximum recovery of platelets or WBCs. In addition, the effects of whole blood volume, hematocrit and tube geometry on the recovery rate have been investigated, confirming that the magnitudes of maximum recovery rates of platelets and WBCs are strongly affected depending on them. Furthermore, we have shown that the dilution of whole blood, increase of whole blood volume and extension of cross-sectional area of the tube are helpful to significantly increase the recovery rate. In view of the real clinical applications, our predictions can be used as a simple guideline to customize the centrifugal conditions according to the trade-off between practical issues such as the time efficiency and platelet integrity. We believe that the present results will explain the reason why the previously reported optimal conditions show wide scatters, and our model may satisfy the necessity of universal optimal condition to maximize the recovery of blood cells in PRP preparation, and will be provide a theoretical background to develop a specially customized (complex, for example) tube geometry with a enhanced capability of blood cell separation. Since our major goal was to predict the concetrnation of platlets (most important blood cell to determine the quality of PRP), we have used a relatively simple model to predict the WBC concentration. If the clinical role of WBCs in PRP is cleared, however, it would be also important to develop more rigorous model to estimate the WBC concentration in PRP preparation, as a future work.
